# A study of inter-lab and inter-platform agreement of DNA microarray data

**DOI:** 10.1186/1471-2164-6-71

**Published:** 2005-05-11

**Authors:** Huixia Wang, Xuming He, Mark Band, Carole Wilson, Lei Liu

**Affiliations:** 1 Department of Statistics, University of Illinois at Urbana-Champaign, 101 Illini Hall, 725 South Wright Street, Champaign, Illinois 61820, USA; 2 W. M. Keck Center for Comparative and Functional Genomics, University of Illinois at Urbana-Champaign, 1201 W. Gregory Drive, Urbana, Illinois 61801, USA

## Abstract

As gene expression profile data from DNA microarrays accumulate rapidly, there is a natural need to compare data across labs and platforms. Comparisons of microarray data can be quite challenging due to data complexity and variability. Different labs may adopt different technology platforms. One may ask about the degree of agreement we can expect from different labs and different platforms. To address this question, we conducted a study of inter-lab and inter-platform agreement of microarray data across three platforms and three labs. The statistical measures of consistency and agreement used in this paper are the Pearson correlation, intraclass correlation, kappa coefficients, and a measure of intra-transcript correlation. The three platforms used in the present paper were Affymetrix GeneChip, custom cDNA arrays, and custom oligo arrays. Using the within-platform variability as a benchmark, we found that these technology platforms exhibited an acceptable level of agreement, but the agreement between two technologies within the same lab was greater than that between two labs using the same technology. The consistency of replicates in each experiment varies from lab to lab. When there is high consistency among replicates, different technologies show good agreement within and across labs using the same RNA samples. On the other hand, the lab effect, especially when confounded with the RNA sample effect, plays a bigger role than the platform effect on data agreement.

## Background

Diversity of microarray data poses some unique and interesting questions on cross-experiment comparisons and the analysis tools needed for such comparisons. Since the invention of the microarray technology in 1995 [1], statistical methods and data mining techniques specific for microarray data have mushroomed [2], many of which have been packaged into commercial software such as GeneSpring and Spotfire. Such tools are useful for handling individual experiments, including quality control, significance testing, and clustering. However, researchers have questioned whether studies across different labs and technology platforms will have an acceptable level of agreement.

Possible incompatibility of results between similar microarray experiments is a major challenge that needs to be addressed, even though the data produced within a single experiment may be consistent and easy to analyze. Different labs produce microarray data in different ways using different technology platforms, such as Affymetrix GeneChip, spotted cDNA array, and spotted oligo array. Affymetrix GeneChip uses one fluorescent dye while the spotted array uses two fluorescent dyes in the experiments. Direct comparison of raw data obtained from different technologies may not be meaningful. Instead, the final form of the data is often presented as relative expression levels, mostly ratios of intensities, after some statistical treatments including filtering, normalization and model-based estimation. Experiments using different technologies require different protocols for analyzing the raw data to derive the ratios. Scientists have published microarray data in a variety of formats including raw intensities and ratios of intensities. Does it make a difference which technology platform is chosen? Can we make use of the studies from different platforms and labs? To answer these questions and provide some guidance for platform comparisons, we report on a comparative study of three different platforms. The experiment is a simple two-tissue comparison between mouse liver and spleen. We used previously published data sets from two different sources as well as new data sets produced in house. There are several similar studies published in recent years [3-8]. A noticeable difference of this study from earlier ones is that we considered lab as a major factor in the comparison. In addition, we compared three major types of technology platforms, namely Affymetrix GeneChip, spotted cDNA array and spotted long oligo array. This study aims to provide a basis for further development of methodologies for comparing microarray data across different experiments and for the integration of microarray data from different labs.

## Results

### Data collection

As summarized in Table 1, a total of five data sets were either collected from a public source or generated in house. The samples for the experiments were normal mouse liver and spleen RNA, which were purchased from Clonetech (Catalog No. 64042-1 liver; Catalog No. 64044-1 spleen) except for the data set GNF generated by Su et al. [9] at the Genomics Institute of the Novatis Research Foundation. Detailed sample descriptions for the GNF data can be found at . Two data sets were downloaded from the NCBI Gene Expression Omnibus , which were generated by Choi et al. at California Institute of Technology (Cal Tech) using Agilent oligo (GEO accession: GSE334) and cDNA arrays (GEO accession: GSE330), respectively. Two other data sets were generated at the Functional Genomics Unit at the W. M. Keck Center for Comparative and Functional Genomics at the University of Illinois using an in-house printed cDNA mouse array and Affymetrix mouse expression set 430A, and the data sets are available at . Another data set was downloaded from  and it was generated using Affymetrix Murine Genome set U74Av2. The data set names (e.g., KC for the cDNA data set generated at the Keck Center at the University of Illinois) given in Table 1 will be used throughout the paper.

**Table 1:** Summary of data collection

**Data Set**	**Array**	**Genes**	**Sample**	**Replicates**	**Data type**	**Platform**	**Lab**	**Data Source**
KC	CI 15K cDNA	15K	Clonetech	4	Raw intensity	cDNA	Keck	In house
KAV	Affymetrix 430A	23K	Clonetech	2	AV(Bioconductor)	Affymetrix	Keck	In house
KLW	Affymetrix 430A	23K	Clonetech	2	Li and Wong	Affymetrix	Keck	In house
KRMA	Affymetrix 430A	23K	Clonetech	2	RMA	Affymetrix	Keck	In house
CC	Riken16K cDNA by Agilent	16K	Clonetech	3	Raw intensity	cDNA	Cal Tech	NCBI GEO
CO	Riken16K Oligo by Agilent	16K	Clonetech	3	Raw intensity	Oligo	Cal Tech	NCBI GEO
GNF	Affymetrix U74Av2	12K	In house	2	AV(MAS4.0)	Affymetrix	GNF	expression.gnf.org

### Consistency of replicates

**Figure 1:**
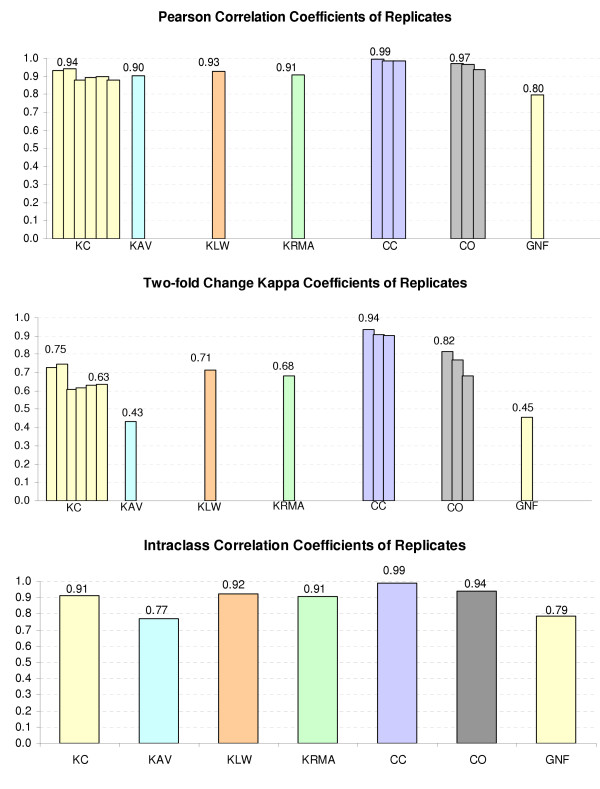
Consistency of replicates.

One indication of data reliability is the consistency of replicates in a particular data set. We used kappa coefficients as well as the Pearson correlation coefficients and intraclass correlation coefficients on the replicates within each data set. Those measures set a benchmark against which the reliability of different platforms can be assessed; see Figure 1. The data set KC has four replicates from double spots of each gene on the array and from the dye swap. Therefore, we can do six pairwise comparisons of replicates. The data sets CC and CO have three replicates each; therefore, there are three pairwise comparisons. All Affymetrix data sets have two replicates and thus only one comparison. From Figure 1, we see that the replicates were quite consistent within each technology. The replicates in all the data sets showed pairwise Pearson correlation coefficients of 0.80 or higher, intraclass correlation coefficients of 0.77 or higher, and kappa coefficients of 0.43 or higher. The data from the Cal Tech (CC and CO) showed the highest agreement among the replicates, and the data from GNF and KAV showed a low level of agreement between replicates. These results suggest a lab effect in microarray data experiments.

For the KC data, we can see that two pairwise comparisons gave slightly higher agreement than the other four. It is, we believe, due to the double spots of each gene on the array. The two comparisons with higher agreement are the comparisons between the replicates within the slides.

### Pairwise comparisons among data sets

**Figure 2:**
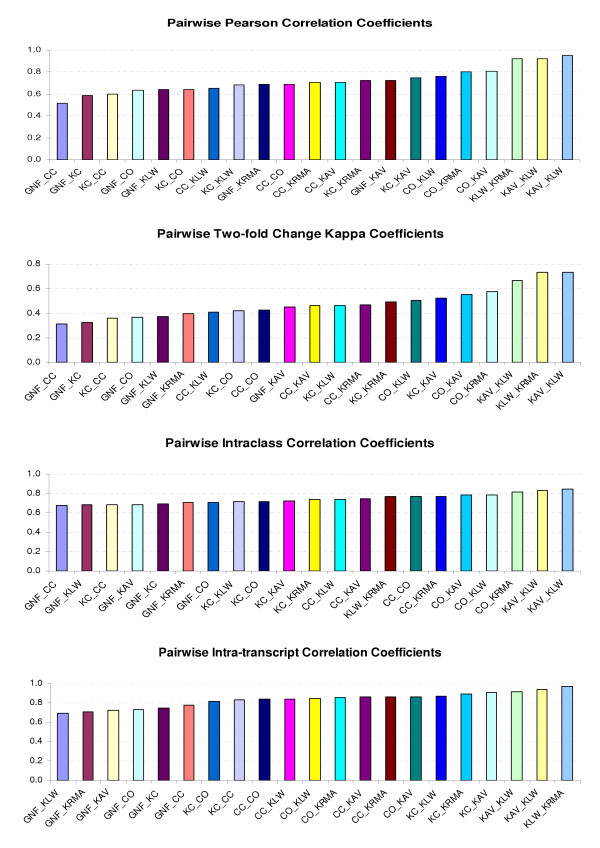
Correlation coefficients for pairwise comparisons between data sets.

Using the matched genes by common UniGene IDs, we compared different data sets in this study. Table 2 shows the Pearson correlation coefficients (PCC), intraclass correlation coefficients (ICC) and intra-transcript correlation coefficients (ITC) on log ratios, and the kappa coefficients (Kappa) for two-fold changes. The detailed descriptions of these measurements can be found in the "Methods" section. Figure 2 shows the histograms of the pairwise PCC, Kappa, ICC and ITC, respectively. With the exception of the GNF, most of the kappa coefficients are between 0.4 and 0.6, as compared to 0.6 for the replicates within the KC data set.

**Table 2:** Correlation coefficients for pairwise comparisons between data sets. Pearson correlation coefficients (PCC), kappa coefficients (Kappa), intraclass correlation coefficients (ICC) and intra-transcript correlation coefficients (ITC) for pairwise comparisons.

Comparisons	No. of Matched Unigene IDs	PCC	Kappa	ICC	ITC
GNF vs. KC	1,838	0.590	0.327	0.693	0.748
GNF vs. CC	1,374	0.513	0.312	0.678	0.774
GNF vs. CO	1,914	0.633	0.365	0.707	0.729
GNF vs. KAV	2,058	0.727	0.452	0.686	0.724
GNF vs. KLW	3,295	0.640	0.374	0.681	0.690
GNF vs. KRMA	3,452	0.686	0.400	0.706	0.705
KC vs. CC	2,730	0.597	0.363	0.681	0.830
KC vs. CO	3,043	0.641	0.423	0.714	0.812
KC vs. KAV	2,964	0.747	0.523	0.726	0.908
KC vs. KLW	4,362	0.680	0.461	0.714	0.868
KC vs. KRMA	4,516	0.725	0.493	0.736	0.893
CC vs. CO	3,262	0.688	0.429	0.770	0.836
CC vs. KAV	2,285	0.708	0.461	0.746	0.859
CC vs. KLW	3,658	0.650	0.407	0.739	0.837
CC vs. KRMA	3,843	0.707	0.472	0.772	0.862
CO vs. KAV	3,001	0.806	0.555	0.781	0.865
CO vs. KLW	4,725	0.759	0.503	0.782	0.847
CO vs. KRMA	5,018	0.805	0.580	0.813	0.854
KAV vs. KLW	7,181	0.923	0.666	0.832	0.917
KAV vs. KLW	7,237	0.955	0.734	0.848	0.938
KLW vs. KRMA	14,130	0.921	0.732	0.765	0.971

From Figure 2 and Table 2, we see that the rankings of the pairwise comparisons are almost the same across four different measures of agreement. All the measures involving GNF are at the low end of the comparisons. The same is observed from the sensitivity check, which was done by leaving out one data set at a time and recording the changes of ICC as shown in Table 3. Excluding GNF resulted in the largest increase in ICC.

**Table 3:** Sensitivity check of the overall comparison among all the data sets

	ICC
Before leaving out	0.662
Leave out GNF	0.703
leave out KLW	0.650
leave out KC	0.663
leave out CC	0.684
leave out CO	0.670

In a recent study, Jarvinen et al. [4] compared Affymetrix GeneChip, commercial cDNA array and a custom cDNA array using the same RNA samples from human cancer cell lines. They found that the data were more consistent between two commercial platforms and less consistent between custom arrays and commercial arrays. Their conclusion is consistent with our findings. In our study, KC is a custom cDNA array, whereas CC and CO are commercial cDNA and oligo arrays, respectively. If we do not consider comparisons involving GNF, we found that KC_CC (shorthand for KC versus CC) and KC_CO comparisons were ranked at the bottom. The samples used in KC, CC, and CO were identical, so biological variability is not an issue here. The variability among those data sets was mainly due to technical factors, such as platforms and labs conducting the experiments. Jarvinen et al. [4] analyzed the experiments conducted in one lab, and therefore their study was mostly concerned with the platform difference. Another study by Culhane et al. [3] used the co-inertia analysis to compare overall expression profiles across different platforms. Their analysis could be used on matched genes, as well as on all the data from different platforms. While they considered the agreement for the overall expression profiles, we focused on the agreement in expressions at the gene level. Using the within-platform variability as a benchmark, we found that these technology platforms exhibited an acceptable level of agreement. For example, the overall comparison of all five data sets using KLW for the in-house Affymetrix gives ICC = 0.662, as compared to the ICC around 0.8 for replicates within each data set. These results indicate that the agreement of different technologies is decent.

**Figure 3:**
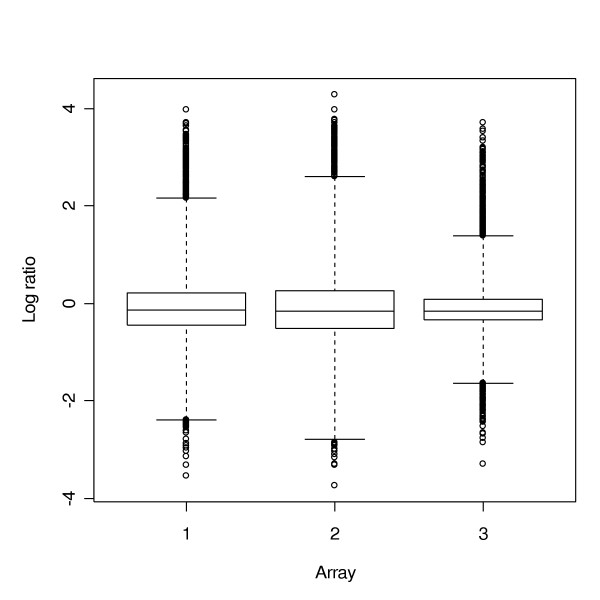
Boxplot of the full data set of CO, with 7,282 Unigene IDs.

**Figure 4:**
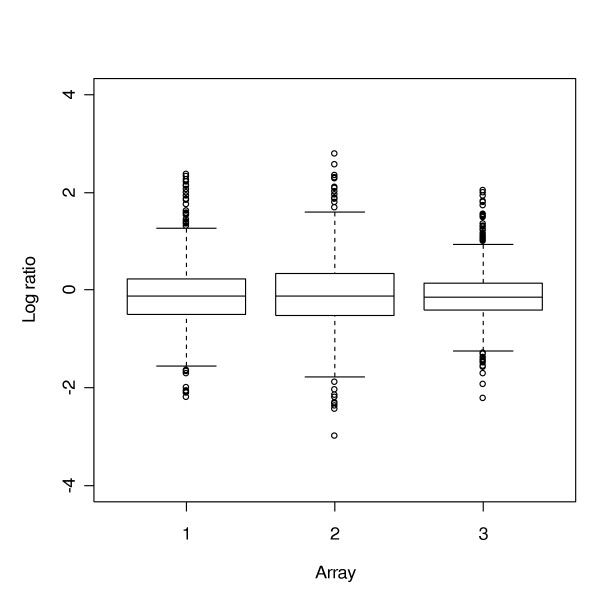
Boxplot of the subset of CO, overlapped with the other 4 datasets, with 551 Unigene IDs.

Since we only used a subset of genes in the study due to the gene matching problem, we asked whether the gene-to-gene variation was impacted by the choice of the subset. A comparison of the box plots for the full data set and for the subset used in the overall comparison showed that the variation in the subset of genes is similar to that of the full data set. For example, Figure 3 and Figure 4 give the box plots for the full data set and the subset of CO, respectively. Therefore, we believe that a reasonable conclusion can be made based on the subset being used.

## Discussion

UniGene has been widely used to match genes on different microarrays. Using UniGene and sequence similarity, Thompson, et al. [10] reported a number of gene markers that showed platform-independent expression profile. When we matched genes from different arrays using the mouse UniGene IDs, we found that there were multiple gene IDs in an array corresponding to one UniGene ID. Those genes were considered as "duplicate" genes, which made the cross matching of genes more complicated. A common approach is to average the expressions of those "duplicate" genes; however, we considered these "duplicate" genes as replicates in the technology and lab comparisons. One observation we should make is that the variability among these "duplicate" genes can be large. For example, in the CC arrays, there are 11,301 genes (upon filtering), corresponding to 8,318 UniGene IDs. Among the 8,318 UniGene IDs, 1,708 of them have "duplicate" genes. For the 12 Unigenes that have 10 or more "duplicate" genes, the ICC of the replicates decreased from 0.99 to 0.86 due to gene matching by Unigene IDs. Note that gene matching was performed only for the comparisons across data sets, and that we did not use the UniGene IDs in measuring the consistency within the same data set. This means that the agreement measures we obtained across different data sets were expected to be slightly lower than those from the replicates, even if the actual agreement was the same within or between data sets. In earlier studies, such as the one reported by Jarvinen et al., it has been shown that using different clones on different arrays is a major factor for the discrepancies between platforms. Using UniGene IDs to match the clones on different platforms can be problematic and result in biased comparisons. Sequence validation of the clones in different arrays may help resolve some of the problems and make the data from different platforms and labs more comparable.

From the present study, we showed that the GNF data set had the lowest agreement with the other data sets. This difference is compounded with the facts that the consistency of replicates within the GNF data set is the lowest and the sample used to generate the data is different from those used by other labs. We believe that the technology platform plays a relatively minor role in the disagreement, but the variation introduced by sample differences is one of the major factors. It has been shown that the expression level can vary significantly between genetically identical mice [11]. Variation among different individuals can be a significant factor for sample differences. Our analysis also indicates that data generated from different labs may have different quality even among the replicates, and thus quality control is important.

**Figure 5:**
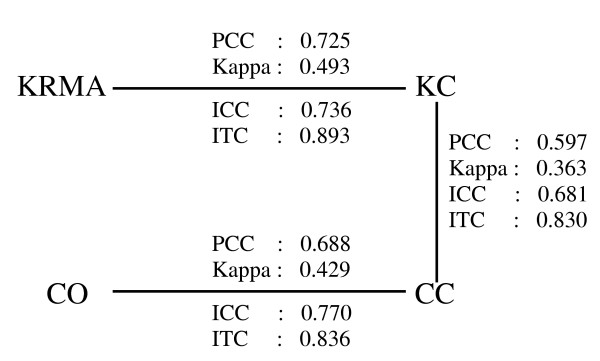
Comparisons between the same technology but different labs (KC_CC) and comparisons between different technologies in the same lab (KRMA_KC and CO_CC).

In this study, we also found that the lab effect can be greater than the platform effect. As shown in Figure 5, the comparisons between two different technology platforms in the same lab (KC_KRMA and CC_CO) showed better agreement than between two labs using the same technology (KC_CC). We also showed that the different summarization methods for Affymetrix exhibited good agreement.

Obviously, the present study has limitations. The results were generated from a very limited number of data sets. Using UniGene IDs for gene matching across data sets can also be questioned. Further research is clearly needed to address these limitations.

## Conclusion

In this paper, we aim to address several issues in comparing microarray data across different platforms and different labs. We demonstrated that the consistency of replicates in each experiment varied from lab to lab. With high consistency among replicates, different technologies seemed to show good agreement within and across labs using the same RNA samples. A closer look at the results indicated that the variability between two labs using the same technology was higher than that between two technologies within the same lab. The source of RNA samples can make a difference in microarray data, however in our present study we do not show conclusive results pertaining to possible sample or lab effects, because we did not have data collected from two different samples within one lab.

## Methods

### Data processing

For the spotted arrays (KC, CC, and CO), we used raw intensity data from both Cy5 and Cy3. We filtered out the non-expressive data points using median plus three times median absolute deviation (MAD, [12]) of the negative control genes as a criterion. We then performed global lowess normalization on each slide. For the KC data, we also performed paired-slide normalization following the method in Yang et al. [13] because of the dye swap in the experiment.

For the in-house Affymetrix data, we used three summarization methods to generate the probe set level signals. KAV and KRMA are based on the R package *affy *of Bioconductor using the average difference (AV) between Perfect Match (PM) and Mis-Match (MM) probe pairs, and the Robust Multi-Array Average (RMA) expression measure developed by Irizarry et al. [14], and KLW is the model-based expression indexes developed by Li and Wong [15]. The GNF Affymetrix data were available to us only in the format of average differences. For both GNF and in-house Affymetrix probe set signals, we performed global lowess normalization for the three pairwise combinations of the liver and spleen slides, and used the averaged lowess adjustment for the normalization. The genes with probe set signals lower than 20 in either liver or spleen tissue were filtered out.

To stabilize the variances of data across the full range of gene expressions, we also performed the generalized log transformation for all the data sets following Durbin et al. [16].

### Gene matching across arrays

There are five different data sets in this study. The origins of the genes vary in those datasets. In order to study inter-lab agreement, we have to first identify common genes represented in different arrays. Based on the annotation of each data set, we found that we could maximize the number of cross-matched genes using the mouse UniGene IDs (Build 107). Based on the common UniGene IDs, we found 551 common genes across all five different data sets. But in the pairwise comparisons, the number of common genes ranged from 1,374 to 5,018 (see Table 2). All comparisons between data sets were made from the matched genes.

### Statistical procedures for inter-platform and inter-lab comparisons

In the analyses, the ratio is defined as normalized and transformed intensity from liver samples versus that from spleen samples.

#### Agreement of two-fold changes using kappa coefficients

An intuitive measurement of agreement is to count the percentage of genes falling in the same categories (two-fold up-regulated, no change, and two-fold down-regulated). However, this percentage can be high even if the data obtained from different platforms are not so compatible. Usually the ratios for the great majority of genes do not show a two-fold change, and the percentage of agreement can be high just due to chance. To adjust for this excess agreement expected by chance, we prefer to use the kappa coefficient, which is a popular measure of inter-rater agreement in many other areas of science. The kappa coefficient was first proposed by Cohen [17] for analysing dichotomous responses, and was extended later to more than two categories of responses. We applied this measure to three categories (two-fold up-regulated, no change, and two-fold down-regulated), and computed the kappa coefficients between two data sets from 3 by 3 frequency tables. For a study of *q *categories, the kappa coefficient is calculated by: , where  is the overall agreement probability,  is the measure of the likelihood of agreement by chance, and *n*_*ij *_is the number of subjects in the (*i*, *j*) cell, *n*_*i*+ _is the sum of the *i *th row, *n*_+ *j *_is the sum of the *j *th column, and *n *is the total number of subjects.

For example, the kappa coefficient between KC and KAV is 0.523. Table 4 gives the two-fold gene regulation frequency table between KC and KAV. Except for the 8 genes that showed two-fold up-regulation in one data set but two-fold down-regulation in another, KC and KAV agreed very well.

**Table 4:** Frequency table for KAV and KC

KAV	KC			
Frequency	-2	0	2	Total
-2	173	136	5	314
0	157	1,972	146	2,275
2	3	112	260	375
Total	333	220	411	2,964

Kappa coefficient = 0.523

#### Correlation coefficients of the ratios

We used three measures of correlation to compare the ratios from different data sets: Pearson correlation coefficient, intraclass correlation coefficient (ICC) and intra-transcript correlation coefficient (ITC). ICC measures the inter-rater reliability relative to the total variability of the ratios. Here, a rater could be a replicate or a technology platform. ICC is the variance of different ratios between UniGene IDs,, divided by the total variance. A high ICC (close to 1) means that the inter-rater ratios vary little relative to the overall variability in the data. In computing the ICC for the replicates,  equals , where  is the variance within UniGene IDs. If we consider lab as a random effect in the overall comparison, the total variance  will equal , where  is the variance between labs. The ICC incorporates both the association between raters and the rater differences, while the Pearson correlation is insensitive to the latter.

We introduced the ITC for pairwise comparisons as described below. For each gene *i*, we defined ρ_*i *_to be the square root of the ratio of within dataset sum of squares (SSW) and the total sum of squares (TSS). A common SSW was used in comparing lab pairs to avoid the problem of having seemingly higher ITC's due to unusually large within-lab variability at some lab. We applied logit transformation to each ρ_*i *_to get γ_*i*_, and then calculated the average γ. Converting γ back to the correlation scale, we obtained .

## Authors' contributions

HW and LL collected data from public resources. MB and CW generated in-house data. HW and LL processed the collected data. HW and XH did statistical analyses. All authors read and approved the manuscript.
